# Pharmacogenomics of Cancer Pain Treatment Outcomes in Asian Populations: A Review

**DOI:** 10.3390/jpm12111927

**Published:** 2022-11-18

**Authors:** Shobha Elizabeth Satkunananthan, Vijayaprakash Suppiah, Gaik-Theng Toh, Hui-Yin Yow

**Affiliations:** 1 School of Pharmacy, Faculty of Health and Medical Sciences, Taylor’s University, Subang Jaya 47500, Malaysia; 2 Clinical and Health Sciences, University of South Australia, Adelaide, SA 5000, Australia; 3 Australian Centre for Precision Health, University of South Australia, Adelaide, SA 5000, Australia; 4 School of Medicine, Faculty of Health and Medical Sciences, Centre for Drug Discovery and Molecular Pharmacology, Taylor’s University, Subang Jaya 47500, Malaysia; 5 Department of Pharmaceutical Life Sciences, Faculty of Pharmacy, Universiti Malaya, Kuala Lumpur 50603, Malaysia

**Keywords:** opioid, cancer pain, gene polymorphisms

## Abstract

In advanced cancer, pain is a poor prognostic factor, significantly impacting patients’ quality of life. It has been shown that up to 30% of cancer patients in Southeast Asian countries may receive inadequate analgesia from opioid therapy. This significant under-management of cancer pain is largely due to the inter-individual variability in opioid dosage and relative efficacy of available opioids, leading to unpredictable clinical responses to opioid treatment. Single nucleotide polymorphisms (SNPs) cause the variability in opioid treatment outcomes, yet their association in Asian populations remains unclear. Therefore, this review aimed to evaluate the association of SNPs with variability in opioid treatment responses in Asian populations. A literature search was conducted in Medline and Embase databases and included primary studies investigating the association of SNPs in opioid treatment outcomes, namely pharmacokinetics, opioid dose requirements, and pain control among Asian cancer patients. The results show that CYP2D6*10 has the most clinical relevance in tramadol treatment. Other SNPs such as rs7439366 (*UGT2B7*), rs1641025 (*ABAT*) and rs1718125 (*P2RX7*) though significant have limited pharmacogenetic implications due to insufficient evidence. *OPRM1* rs1799971, *COMT* rs4680 and *ABCB1* (rs1045642, rs1128503, and rs2032582) need to be further explored in future for relevance in Asian populations.

## 1. Introduction

Pain is the most prevalent symptom experienced by patients with advanced cancer. Affecting up to 96% of patients [1], cancer pain significantly impacts patients’ quality of life and is a poor prognostic factor amongst patients with advanced cancer. Globally, inadequate pain relief continues to be a serious health concern for cancer patients with pain. It is estimated that more than 30% of patients worldwide receive inadequate pain relief [1]. The prevalence of undertreated cancer pain in Asia is exceptionally high at as much as 59%, compared with 40% and 39% in Europe and the United States, respectively [2]. A study reported that there may be a significant under-management of cancer pain due to inadequate use of opioid analgesics in all six Southeast Asian countries [3]. In 2015, the average morphine equivalence in the Southeast Asian region was just 1.7 mg per capita, when the global average was 61.5 mg per capita [4]

Since the introduction of the three-step analgesic ladder by the World Health Organization (WHO) [5], strong opioids such as morphine have been the mainstay of analgesic therapy in the management of moderate-to-severe cancer pain [6]. A prospective study in a Malaysian palliative care unit observed that of the 61.1% of hospitalized cancer patients who reported having pain, with up to 82% had moderate to severe pain [7]. Although morphine is most widely prescribed in clinical settings, appreciable inter-individual differences in its effectiveness is a major disadvantage for its clinical use [8]. Most patients taking morphine for cancer pain achieve good analgesia with minimal side effects [9]. However, up to 30% of patients could experience inadequate pain relief despite escalating doses and/or experience intolerable side effects [10].

Clinical evidence shows that patients need variable opioid doses, and that the relative efficacy of available opioids varies between patients [11,12]. Several factors such as variable bioavailability [13,14] and differences in the intensity of pain stimuli and perception have been suggested as possible reasons for this inter-individual variability [15,16,17]. Additionally, pharmacogenetics studies have tried to provide a reasonable explanation for the inter-individual variability observed in analgesic response. This is especially crucial for long-term opioid use as genetic variations have been shown to impact cellular responses elicited by opioid stimulation [18].

Previous studies have identified genetic polymorphisms in drug-metabolizing enzymes (*CYP2D6*, *CYP3A4*, *CYP3A5*), membrane drug transport proteins (*ABCB1*), opioid receptors (*OPRM1*), and pain receptors (*COMT*) associated with the efficacy, dose, and toxicity of opioids [19,20]. However, not all genetic associations have been replicated in subsequent studies, further complicating the situation. The largest study to date, the European Pharmacogenetic Opioid Study (EPOS) with 2294 European cancer patients, did not find any association between opioid dose and 112 SNPs in 25 candidate genes [18]. Even for the most extensively studied *OPRM1* A118G SNP, a recent meta-analysis reported statistical significance in Asian patients only [21].

Therefore, this review aimed to determine the association of genetic variants, specifically SNPs, with variability observed in treatment responses in Asian patients treated with opioids for cancer pain. This review includes original pharmacogenomics studies that have investigated treatment outcomes of morphine, fentanyl, oxycodone, tramadol, codeine, hydrocodone, hydromorphone, levorphanol, methadone, and oxymorphone in the management of cancer pain in Asian patients.

## 2. Materials and Methods

The literature search was conducted in two databases, Medline and Embase using free-text and MeSH terms. The Boolean operator “OR” was used to group search terms into four main subject groups. The first subject group covered terms relating to cancer pain, including ‘cancer pain’, or ‘malignant’, or MeSH ‘cancer pain’, or MeSH ‘malignant’. The second subject group covered terms relating to pharmacogenetic, including ‘gene polymorphism*’; or: ‘Single Nucleotide Polymorphism*’; “pharmacogen*”; or “genetic*”; or “genomic*”, or “genotype” or MeSH “pharmacogenetics”. The third subject group covered terms relating to opioids, including “opioid*”, or “opiate*”, or “morphine”. The fourth subject group covered terms relating to treatment outcome, including “treatment outcome*”, or “treatment response*”, or “response*”, or “efficacy”, or “pain control”. Each group of search terms was searched simultaneously with the “AND” Boolean operator to identify original research articles published up to November 2020 that investigated the association of SNPs with opioid treatment outcomes in cancer patients. The search was limited to articles published in English with human subjects. Abstracts or conference proceedings were included if the content was not published before.

**Figure 1:**
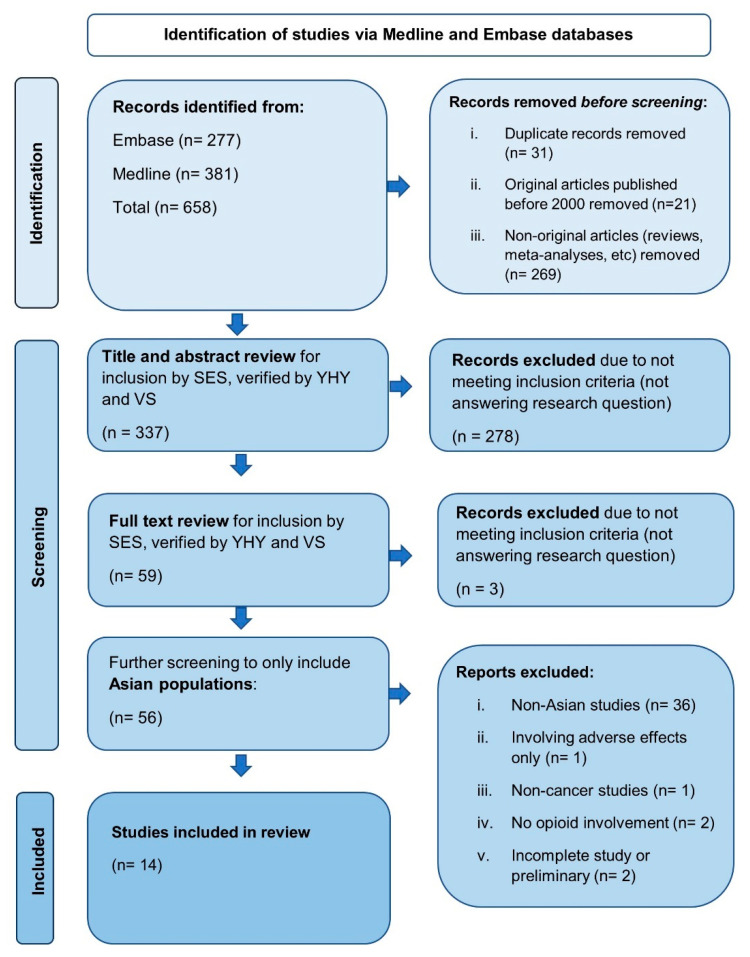
Flow chart of the selection process and search strategy for this study.

A total of 381 papers from Medline and 277 papers from Embase were identified. The 658 records were screened for original articles published between 2000 to November 2020. After removing duplicates, reviews and meta-analyses published before 2000, a total of 337 papers were shortlisted. The title and abstract of each manuscript were reviewed against the inclusion and exclusion criteria by researcher SES, and independently verified by YHY and VS. From this, 281 articles that involved animal studies, papers not investigating pharmacogenetic associations, and studies that did not involve genotyping participants were excluded. The remaining 56 studies were screened for studies involving non-Asian patients and those that did not involve cancer patients or treatment with opioids. Reference lists of past reviews were checked for other papers of interest to ensure that the search was all inclusive. This resulted in 14 articles fulfilling the inclusion criteria and were included in this review (Figure 1).

Pain control was assessed as changes in pain scores. The analgesic effect seen in opioid consumption was assessed based on administered dose (mg/day), route of administration, and frequency of administration. The findings in this review have been stratified and presented according to the opioids of interest.

## 3. Results

**Figure 2:**
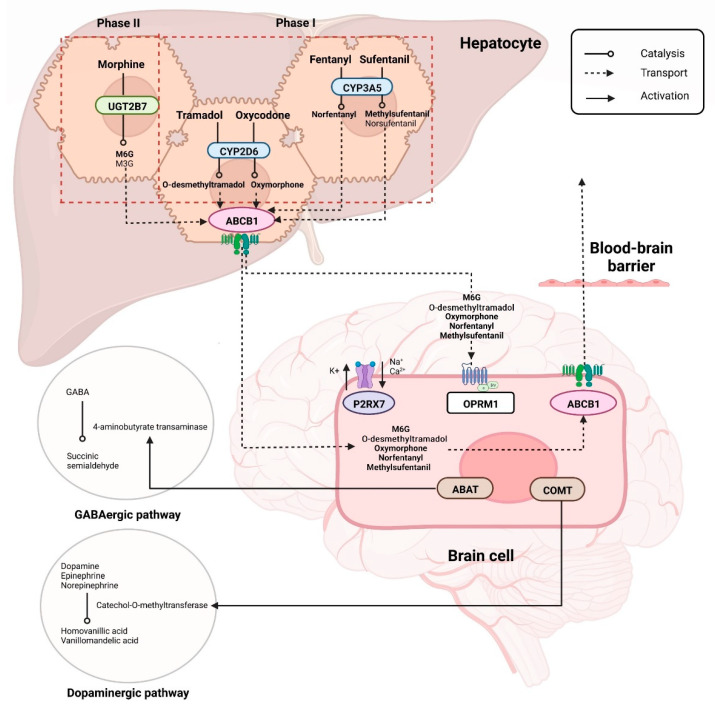
Pathways of genes that affect cancer pain treatment outcomes via drug-metabolizing enzymes (*CYP2D6*, *CYP3A5*, *UGT2B7*), opioid receptor (*OPRM1*), pain modulation through GABAergic and dopaminergic pathways via neurotransmitter-metabolizing enzymes (*COMT*, *ABAT*), efflux drug transporters (*ABCB1*) and cell depolarization via ion channels (*P2RX7*). Created with BioRender.com.

A total of 17 SNPs from eight genes with significant associations with opioid dose requirements, pharmacokinetics, and pain control were identified from the included studies (Table 1). The combined pathways of these genes, which are involved in phase I and phase II opioid metabolism, opioid receptor binding, neurotransmitter metabolism, drug transport, and ion channel, were analyzed for associations with treatment outcomes (Figure 2).

### 3.1. Phase I Opioid Metabolism: Cytochrome P450 Enzyme-Coding Genes

The cytochrome P450 proteins are monooxygenases, which catalyze many reactions involved in the metabolism of opioids such as codeine, hydrocodone, oxycodone, methadone, tramadol, fentanyl; and synthesis of cholesterol, steroids and other lipids [36].

#### 3.1.1. CYP2D6

*CYP2D6* encodes a member of the cytochrome P450 superfamily of enzymes. The *CYP2D6* gene is highly polymorphic, with over 135 distinct functional allelic variants resulting in considerable variation in enzymatic activity in the general population [37,38]. A deficiency of the *CYP2D6* enzyme is inherited as an autosomal recessive trait and those with this deficiency are classified as poor metabolizers. Among the other metabolizer phenotypes, enzyme activity is highly variable, from extremely high in ultrarapid metabolizers to markedly reduced in intermediate metabolizers [37].

- Tramadol

The association of *CYP2D6* genotypes with the dose of tramadol consumed within 48 h was explored in a prospective study conducted among 70 Chinese gastric cancer patients treated with tramadol post-gastrectomy [22]. In this study, the patients were categorized into three groups according to their *CYP2D6* genotypes: those without the CYP2D6*10 allele (group I, n = 17), patients heterozygous for CYP2D6*10 (group II, n = 26), and patients homozygous for CYP2D6*10 (group III, n = 20). The study found that the CYP2D6*10 allele had a significant impact on analgesia, as the dose of tramadol consumed at three time points, 4, 24 and 48 by patients in group III was significantly higher than that in groups I or II (*p* < 0.05) [22].

Tanaka et al. evaluated the impact of *CYP2D6* genotypes on the plasma concentrations of tramadol and its demethylated metabolites and drug tolerability among 70 Japanese cancer patients [24]. Although the patients were grouped according to their phenotypes (Table 2), the authors found that the *CYP2D6* genotype did not affect the plasma tramadol concentration [24].

However, the plasma concentration of O-desmethyltramadol and its ratio to tramadol were lower in the *CYP2D6* intermediate and poor metabolizer (IM + PM) groups compared to normal metabolizers (NM) (Table 3) [24]. The plasma concentration of N-desmethyltramadol and its ratio to tramadol were higher in the IM + PM groups than the NM group (*p* = 0.001 and *p* = 0.001, respectively) [24]. This study also showed that CYP2B6*6 and CYP3A5*3 polymorphisms did not affect the plasma concentrations of tramadol and its demethylated metabolites [24].

- Fentanyl

The study by Wu et al. also explored the impact of CYP2D6*10 polymorphism on 207 Chinese gastric cancer patients, particularly those treated with fentanyl after undergoing radical gastrectomy [23]. The cumulative amount of fentanyl consumption significantly increased among patients homozygous for the mutant CYP2D6*10 (MM) genotype at 6, 12, and 24 h postoperatively, compared with the wild type CYP2D6*1 (WW) genotype (*p* < 0.05) (Table 4). In addition, the visual analog scale (VAS) score in the MM group was also significantly higher than the WW group in the analepsis period after general anesthesia and at 6 h postoperatively (*p* < 0.05) (Table 5) [23].

#### 3.1.2. CYP3A5

The human CYP3A subfamily, consisting of *CYP3A4*, *CYP3A5*, *CYP3A7* and *CYP3A43*, is one of the most versatile biotransformation systems that facilitate the elimination of drugs. *CYP3A4* and *CYP3A5* together account for approximately 30% of hepatic cytochrome P450, and approximately half of medications that are oxidatively metabolized by P450 are CYP3A substrates. Both *CYP3A4* and *CYP3A5* are expressed in the liver and intestine, with CYP3A5 being the predominant form expressed in extrahepatic tissues [39].

Takashina et al. investigated the impact of *CYP3A5* on fentanyl pharmacokinetics among 60 Japanese cancer patients undergoing conversion to a transdermal fentanyl from a previous treatment of either oral morphine or oxycodone [25]. In this study, the plasma concentration of fentanyl normalized with the measured absorption rate was significantly higher in those carrying the CYP3A5*3*3 genotype than in those with the *1*1 and *1*3 genotypes [25].

### 3.2. Phase II Opioid Metabolism: UGT Enzyme-Coding Genes

#### UGT2B7

*UGT2B7* encodes for UDP-Glucuronosyltransferase-2B7 phase II metabolism isoenzyme. Morphine is primarily metabolized by the hepatic enzyme *UGT2B7* to two active metabolites morphine-3-glucuronide (M3G) and morphine-6 glucuronide (M6G) [40].

- Morphine

The relationship between SNPs in *UGT2B7* and the efficacy of morphine treatment in cancer pain was investigated in 120 Chinese cancer patients [26]. Morphine was administered via patient-controlled infusion pumps. The VAS score was used for pain assessment at 0.5, 4, 6, 12-, 24-, 48-, and 72-h post-morphine treatment. For the C802T (rs7439366) SNP in *UGT2B7*, the plasma concentration of morphine for patients with the CC genotype was significantly lower than that in patients carrying the CT or TT genotypes (*p* < 0.05). The VAS score of patients with either CT or TT genotypes was significantly higher than those with CC genotype (*p* < 0.05) [26]. G221T, another polymorphism in *UGT2B7* was also analyzed in this study. Carriers of the G allele (patients with GG and GT genotypes) had no significant difference in pain scores [26].

- Oxycodone

Li et al. investigated the impact of *UGT2B7* C802T (rs7439366) on pain relief in 47 Han Chinese patients with malignant tumors receiving prolonged-release oxycodone [27]. Patients were grouped into either the refractory group, which indicated significant pain interference, or the remission group, which indicated no significant pain interference. Even though the overall carriage of the T allele (CT and TT genotypes) was 25% in the whole cohort, it was significantly higher in the refractory group (32.4%) compared to the remission group (21.3%) (*p* = 0.047). The authors also found that there were more patients with TT genotype in the refractory group (23.5%) compared to the remission group (2.5%)(60%) (*p* < 0.05) [27].

### 3.3. Opioid Receptor Gene

#### OPRM1

*OPRM1* encodes for the mu (μ) opioid receptor. *OPRM1* is the primary target of both endogenous and exogenous opiates including morphine and tramadol and has been shown to mediate both baseline nociception and response to *OPRM1* agonists [41].

- Morphine

The added value of determining *OPRM1* SNP as a genetic factor in the optimization of cancer pain treatment was evaluated by Hajj et al. [28]. A total of 89 Lebanese cancer patients treated with continuous intravenous injection of morphine in this study required high varied morphine doses: 7 to 210 mg per 24 h with mean doses of 34.78 ± 33.26 mg. The mean dose of morphine significantly decreased with age (*p* = 0.043) but increased with the duration of morphine treatment (*p* = 0.029). Patients carrying the AG genotype for *OPRM1* c.118A > G (rs1799971) required significantly higher morphine doses than patients with the AA genotype (*p* < 0.001) [28].

- Tramadol/Paracetamol combination

In 2011, Liu et al. explored the efficacy of tramadol/paracetamol combination in 96 Chinese patients with adenocarcinoma of the colon, rectum, or stomach and the *OPRM1* A118G (rs1799971) polymorphism [29]. Compared with the AA genotype (wild type), patients carrying the G allele (AG or GG genotypes) had a significantly reduced response to treatment. The pre- and post-treatment VAS scores for patients carrying the G allele were 3.1 and 2.6, respectively compared to patients with AA genotype with pre-treatment and post-treatment VAS scores of 3.0 and 0.9, respectively (*p* <0.001) [29].

- Sufentanil

The effects of *OPRM1* SNPs on the analgesic effect and consumption of sufentanil after thoracoscopic-assisted radical resection of lung cancer were evaluated in 225 Han Chinese cancer patients [30]. The authors found that rs1799971 and rs1323040 were associated with the analgesic effect and dose of sufentanil consumed by the patients. The doses of sufentanil consumed by double heterozygous patients for both SNPs at 6, 24 and 48 h were significantly higher than those consumed by patients who were wild type for both SNPs [30]. However, this study also found that there was no significant difference in sufentanil doses and patients’ VAS scores regardless of their genotypes for the rs563649 SNP in *OPRM1* at three different time points (both *p*> 0.05) [30].

### 3.4. Neurotransmitter-Metabolizing Enzyme-Coding Genes

#### 3.4.1. COMT

*COMT* encodes for the catechol-O-methyltransferase (COMT) enzyme which is responsible for catalyzing the transfer of a methyl group from S-adenosylmethionine to catecholamines, including neurotransmitters dopamine, epinephrine, and norepinephrine. In addition, *COMT* is known to be involved in pain modulation, through dopamine-mediated change in enkephalins neuronal content [42], followed by a compensatory regulation of μ-opioid receptors in various regions of the brain [42,43].

- Morphine

The effects of the G472A (rs4680) SNP in *COMT* on plasma concentration and dose requirements of morphine were evaluated in a prospective study conducted in 48 Japanese patients with cancer [31]. The authors found that the plasma concentration and the required dose of morphine were significantly lower for patients with AA genotype of this SNP compared to carriers of the G allele (AG and GG genotypes) on day 1 (*p* = 0.008 and 0.03, respectively). However, this significance was lost on day 8 of the treatment [31].

The significant difference was later replicated by the same group in a later study [32]. This time the average morphine dose was significantly higher for patients with the GG genotype when compared to carriers of the A allele (AA and AG genotypes) on day 1 of their treatment (35.2 ± 11.5 mg, 29.5 ± 2.3 mg, and 25.0 ± 7.1 mg respectively) (*p* = 0.013) [32]. However, the pain numerical rating score before and after treatment did not differ between these two groups [32]. Interestingly, on day 1, dose titration, which was performed to reach a numerical rating score of ≤3 and pain control ≥33%, was successful in 76% of all cases, but unsuccessful in 60% of patients with the GG genotype [32].

#### 3.4.2. ABAT

*ABAT* codes for the γ-Aminobutyric acid (GABA)-transaminase enzyme, which is responsible for the metabolism of inhibitory neurotransmitter GABA. In addition, GABA neurons and receptors are found in supraspinal sites known to coordinate the perception and response to painful stimuli and this system has been shown to regulate sensory information processing in the spinal cord [44,45].

- Multiple opioids

The rs1641025 SNP in *ABAT* was significantly associated with opioid responsiveness in cancer pain [33]. The authors found that those carrying the CC genotype required the least amount of opioid compared to the CT and TT genotypes before and after increasing opioid dosage (*p* < 0.001). Additionally, the CT genotype was found to have the lowest mean pain severity after the increment in the opioid dose (*p* < 0.001) [33].

### 3.5. Drug Transporter Gene

#### ABCB1

The protein encoded by this gene, P-glycoprotein (P-gp), is an ATP-dependent drug efflux pump for xenobiotic compounds with broad substrate specificity. It is responsible for decreased drug accumulation in multidrug-resistant cells and often mediates the development of resistance to anticancer drugs [46].

- Fentanyl
  Takashina et al. investigated the impact of *ABCB1* 1236TT (rs1128503) on fentanyl pharmacokinetics among 60 Japanese cancer patients undergoing conversion to transdermal fentanyl from previous treatment of either oral morphine or oxycodone [25]. They showed that rescue medication was needed by fewer patients with the TT genotype compared to the other genotypes (*p* = 0.036) [25].
- Sufentanil
  A study conducted among 225 Han Chinese patients with lung cancer reported the association of *ABCB1* SNPs, rs2032582 and rs1128503 with the analgesic effect and dose of sufentanil taken for pain relief [30]. The doses of sufentanil required by double heterozygous patients at 6, 24 and 48 h were significantly higher than those consumed by patients who were wild type for both SNPs [30]. However, another SNP, wild type rs1045642 in *ABCB1* did not show any significance (*p* > 0.05). There were no significant differences in the VAS scores at the three time points nor association with adverse effects [30].
- Multiple opioids
  Gong et al. evaluated the influence of *ABCB1* C3435T (rs1045642) polymorphism on opioid requirements among 112 Chinese patients [34]. In this study, morphine, tramadol, sustained-release morphine, oxycodone, transdermal fentanyl and paracetamol were dosed according to the intensity of the cancer pain. The authors reported that compared with CC/CT genotypes, patients homozygous for the T allele received higher 24 h- and weight-surface area-adjusted-24 h- opioids doses (*p* = 0.057 and 0.028, respectively) [34].

### 3.6. Ion Channel Gene

#### P2RX7

The purinergic receptor P2X, ligand-gated ion channel 7, *P2RX7*, encodes the purinergic receptor P2X7 which is a ligand-gated cation channel that opens in response to ATP binding and leads to cell depolarization. *P2X7* is expressed in peripheral and central nervous systems and the immune system, mediating and modulating pain. *P2X7* function has been previously linked to chronic inflammatory and neuropathic pain [47].

- Fentanyl
  The correlation of rs1718125 in *P2RX7* with postoperative fentanyl analgesia was investigated in an observational study among Han Chinese patients with lung cancer [35]. This SNP was found to be significantly associated with postoperative pain and fentanyl dose (*p* < 0.05). Patients carrying the GA and AA genotypes required more fentanyl doses for pain control within 48 h postoperatively (*p* < 0.05). The postoperative VAS score was also significantly higher in carriers of the GA genotype when compared to the GG genotype group in the period of analepsis after general anesthesia and 6 h post-surgery (*p* = 0.041 and *p* = 0.030, respectively). Meanwhile, the postoperative VAS score was significantly higher in the A homozygotes than in the period of analepsis after general anesthesia (*p* < 0.001), at 6 (*p* = 0.006) and 24 h (*p* = 0.016) post-surgery [35].

## 4. Discussion

The findings of this review have highlighted the role of genetic variants in drug-metabolizing enzymes (*CYP2D6*, *CYP3A5*, *UGT2B7*), neurotransmitter-metabolizing enzymes (*COMT*, *ABAT*), transporters (*ABCB1*) and drug receptor (*OPRM1*) and ion channels (*P2RX7*) in contributing to inter-individual variability in opioid treatment responses in Asian patients with cancer pain.

Genetic polymorphisms in *CYP2D6* are possibly the most well-researched. SNPs in *CYP2D6* contribute to the interindividual variability seen in this enzyme’s activity and thus its metabolizing capacity [48]. The CYP2D6*10 allele has been associated with a higher tramadol dose [22] and lower plasma concentration of O-desmethyltramadol [24] due to a reduced enzyme stability [49]. The CYP2D6*10 variant results in an amino acid substitution from proline (in wild type CYP2D6*1) to serine leading to a decreased enzymatic activity [49]. The reduced formation of the M1 metabolite, which has 200-300 folds higher affinity for the μ-opioid receptor and is six times more potent than tramadol [50,51], leads to an observable reduction in tramadol’s analgesic effects. With a frequency up to 50% in Asian populations, the CYP2D6*10 polymorphism produces an intermediate metabolizer phenotype and is predominant and well-documented among Asian populations [52,53,54].

In the study by Tanaka et al. [24], patients who were IMs and PMs were combined (n = 25) and compared with NMs (n = 45). However, according to the latest Clinical Pharmacogenetics Implementation Consortium (CPIC) guideline, CYP2D6*1/*5 and *2/*5 genotypes have been re-classified from NM to IM phenotype based on their activity scores [53]. Similarly, CYP2D6*5/*14 and *14/*14 have been re-classified from PM to IM phenotype. Based on this update, the carriers of the CYP2D6*1/*5 and *2/*5 genotypes would have to be re-classified from the NM to the IM + PM group [24,53]. As the frequency of the CYP2D6*5 variant has been reported to be relatively low at 6.3% in Japanese populations [55], it is highly unlikely that the distribution of the groups analyzed by Tanaka et al. would change significantly. The findings of lower plasma concentration of the O-desmethyltramadol metabolite in the IM + PM group compared to the NM group may be due to lower *CYP2D6* activity [24]. The higher plasma concentration of the N-desmethyltramadol metabolite in the IM + PM group compared to the NM group may be a result of alternative metabolic pathways, mainly through the *CYP2B6* and *CYP3A* enzymes [24]. Although CYP2D6 is not involved in the metabolism of fentanyl, the CYP2D6*10 variant (T allele) has been associated with higher fentanyl dose and pain scores compared to the wild type CYP2D6*1 (C allele) [23]. Grimsrud and colleagues demonstrated that CYP2D6*9 and *29 alleles were associated with impaired clearance of fentanyl in burn patients [56]. The metabolism of fentanyl has been shown to be predominantly mediated by CYP3A4 and CYP3A5 enzymes [25,57]. It is possible that fentanyl’s metabolism could become dependent on CYP2D6 due to either polypharmacy overwhelming the CYP3A4/5 enzymes or co-administration of CYP3A4/5 inhibitor(s) as approximately half of the medications are metabolized by CYP3A enzymes [39,57].

Polymorphisms in *UGT2B7* are known to affect morphine glucuronidation to form M3G and M6G metabolites [40,58].rs7439366 results in the substitution of tyrosine at position 268 (UGT2B7*1) to histidine (UGT2B7*2). UGT2B7*2 homozygotes are lower in the Chinese population (9.2%) when compared to the Caucasian population (25.3%) (*p* < 0.001) [58,59]. In this review, the C802T (rs7439366) SNP in *UGT2B7* was studied in patients receiving morphine and prolonged-release oxycodone [26,27]. Carriers of the CC genotype had significantly lower plasma concentrations of morphine and pain scores compared to carriers of the T allele (CT or TT genotype) for most time points [26]. Conversely, there were more T homozygotes than C homozygotes in the refractory group receiving prolonged-release oxycodone (with significant pain interference indication) [27]. Although there was no significant difference between carriers of the T allele (CT and TT genotypes), Li et al. speculated that the T allele may play a role in reducing the effectiveness of prolonged-release oxycodone [27].

These results are consistent with previous studies suggesting that the rs7439366*C allele may enhance analgesic efficacy [58,59]. M3G and M6G, which are selective for the μ-opioid receptors, were found at high levels and significantly associated with the rs7439366*C allele [59]. Yang and colleagues previously found that carriers of the mutant *UGT2B7* rs7439366 allele had a higher affinity for M3G and M6G compared to their wild type *UGT2B7* counterparts [58]. Paired with *UGT1A9* polymorphisms, the homozygous mutant *UGT2B7* allele carriers tend to produce M6G, which is known to be more potent than M3G [58,60], and thus having more analgesic effects than those who carry the wild type allele. However, the level of evidence for the association between response to morphine and oxycodone and *UGT2B7* rs7439366*T allele has been listed as insufficient (levels 3 to 4) to be used as a biomarker for treatment response in the PharmGKB database [61].

Exogenous opioids including morphine bind to the μ-opioid receptor to bring about opioid-induced analgesia [62,63]. The most widely studied polymorphism within *OPRM1* is the G472A (rs1799971) SNP, which brings about the substitution of asparagine at position 40 (A allele) to aspartic acid (G allele) [64]. This SNP has particular importance in Asian populations as it occurs at a high frequency of 40-60% relative to a moderate 15% in European populations [65]. The results of this review show that the dose of morphine required for adequate pain relief was higher in rs1799971*AG genotype carriers compared to A homozygotes [29]. Carriers of the G allele (GA and GG genotypes) had significantly reduced treatment response and higher pain scores compared to that of the AA genotype (wild type) [28]. These findings suggest that the mutant G allele may reduce the analgesic efficacy of morphine. Previous studies have demonstrated that this SNP decreased signaling efficacy and probably reduced expression of the μ-opioid receptor, which led to reduced μ-receptor binding potential in the brain, explaining the need for higher doses of morphine [66,67].

Sufentanil, being a fentanyl analog, also binds to the μ-opioid receptor. Among sufentanil-treated patients, both sufentanil dose and analgesic effect were associated with the rs1799971 and rs1323040 in *OPRM1* [43]. The dose requirement of patients heterozygous for both SNPs was higher than wild type homozygotes for both SNPs [43] suggestive of the mutant allele reducing opioid analgesic efficacy [66,67].

COMT is responsible for degrading the majority of the dopamine in the prefrontal cortical regions of the brain [68]. The *COMT* SNP G472A (rs4680) has been widely associated with various neuropsychological phenotypes from the dopaminergic pathway, including pain modulation, hyperalgesia, anxiety disorders, opioid-related disorders, and substance addiction [69]. This SNP changes the amino acid at codon 158 from valine to methionine, resulting in the alteration of the enzyme structure and hence reducing its activity by a quarter [70]. In this review, rs4680 was associated with lower morphine dose the plasma concentration in the AA genotype [31] compared with the GG genotype [32]. Two studies have demonstrated that the G allele carriers have reduced analgesic efficacy and require higher doses of morphine when compared to carriers of the A allele (AG and AA genotypes) [71].

However, these results are contradictory to other studies that have reported the A allele being associated with reduced analgesic efficacy [70,72]. The decreased enzymatic activity in the mutant A allele has been shown to lead to higher levels of dopamine in their prefrontal cortex compared to their wild type G allele counterparts and, consequently, is associated with a lower pain threshold [70,72]. This could be explained by COMT largely affecting pain perception via the dopaminergic pathway but not directly modulating the binding affinity of μ-opioid agonists to μ-opioid receptors. Therefore, the A allele carriers would have a higher sensitivity to pain but require less doses of morphine. However, pain control was not associated with *COMT* rs4680′s genotypes [31,32].

Pooled data from a meta-analysis did not find any statistical difference in opioid consumption in patients carrying *COMT* rs4680 homozygous wild type G allele and mutant A allele in the first 24 h of treatment [73]. However, patients who were heterozygous required significantly lower doses of opioid, compared to those who were G homozygotes [73].

The *ABAT* rs1641025 SNP represents a substitution of wild type T allele to the mutant C allele in the gene encoding for GABA transaminase. In this review, the opioid dose requirement was found to be lowest in patients with homozygous for the ABAT rs1621025*C allele, while pain scores were lowest in patients who were heterozygous for this SNP [33]. These findings are suggestive of the C allele variant being associated with low pain scores and, hence, enhanced opioid efficacy [33]. To date, there has been a lack of studies on this SNP to show its clinical significance. However, pain perception is thought to be modulated in the GABAergic synapse pathway by the degradation of γ-aminobutyric acid (GABA) into succinic semialdehyde by GABA transaminase, consequently deactivating GABAergic transmission [44]. Hence, GABA transaminase modulates GABAergic transmission in the central nervous system and subsequently regulates physiological functions including pain perception. While *ABAT* rs1641025 may not influence the opioid dose requirement directly, the pain perception modulated by this SNP may be associated with a higher pain threshold and thus a lower opioid dose requirement. Further studies are needed to explore the potential pharmacogenetic role this SNP may play in pain control and opioid analgesic responsiveness and possibly its usefulness as a biomarker.

The bioavailability of administered opioids is affected by P-gp, an ATP-binding cassette (ABC) family of efflux transporters encoded by *ABCB1* that will remove drugs [19]. One of the most extensively studied polymorphisms in *ABCB1* is in exon 26, C3435T (rs1045642), while the others are present in exon 21, G2677T (rs2032582) and exon 12, C1236T (rs1128503). Of the three, only rs2032582 SNP results in the change in amino acid (alanine to threonine at position 893). Previous studies have demonstrated that patients homozygous for the mutant rs1045642*T allele display two to three folds lower expression of P-gp relative to the wild type CC genotype [74,75]. Although the exact mechanism of how the T allele of this SNP influences drug transport activity is not well understood, it has been hypothesized that it may result in altered exposure to toxins and drugs, thus reducing the efficacy or toxicity of pharmacotherapeutic agents, including opioids [76].

In the current review, rs1128503, rs2032582 and rs1045642 were studied in patients receiving fentanyl [25], sufentanil [30], and multiple opioids [34]. Among patients receiving fentanyl, rescue medication was needed by fewer patients who were homozygous for the mutant rs1128503*T allele compared to carriers of the C allele (CT and CC genotypes) [25]. This suggests that the mutant T allele may enhance analgesic efficacy, possibly extending to protective effects against adverse effects. The dose of sufentanil in patients who were double heterozygous for rs2032582 and rs1128503 was higher than patients who were wild type G and T homozygotes for these SNPs, respectively [30]. Contrary to findings by Takashina et al. [25], Zhao et al. found that the mutant rs2032582*A and rsrs1128503*T alleles had reduced analgesic efficacy [30], which could be preceded by the reduced expression of *ABCB1* and activity of P-gp [74,75,76]. In a study conducted among patients treated with multiple opioids, the opioid dose requirement was higher in patients homozygous for the mutant rs1045642*T allele when compared to carriers of the wild type rs1045642*C allele [34], which matches the findings of previous studies [74,75,76]. However, sufentanil dose was not significantly different among rs1045642 genotypes [30]. Pain scores were also not significantly different among rs2032582 and rs1045642 genotypes [30].

The function of purinergic receptor *P2X7*, encoded by *P2RX7*, has been linked to chronic inflammatory and neuropathic pain. The expression of *P2X7* receptors is upregulated in both dorsal root ganglia and injured nerves, and monocytes and lymphocytes in patients with neuropathic pain. In animal models of inflammatory and neuropathic pain, P2RX7 disruption reduces hypersensitivity [47]. In this review, the *P2RX7* rs1718125 SNP was significantly associated with postoperative pain and fentanyl dose in patients with lung cancer [35]. Among patients treated with fentanyl, the dose requirement for pain control was highest in A allele homozygotes and that the AA and GA genotype groups had significantly higher pain scores than the GG genotype group [35]. The findings of this study indicate that the A allele reduces the analgesic efficacy of opioids. This contradicts with the findings of a recent study which reported that carriers of the A allele required lower fentanyl and had better pain scores in comparison to the G allele homozygotes [77]. To date, there have been limited studies exploring the association of *P2RX7* polymorphisms and opioid dose requirements, pharmacokinetics, or pain control. The potential pharmacogenetic relevance of this SNP needs to be further investigated as pre-clinical studies have shown that *P2X7* may contribute to pain modulation both by its effect on peripheral tissue and through alterations in central nervous system processing [47].

A vast majority of the studies included in this review had patients of East Asian ancestry (Japanese, Chinese, Taiwanese). As a result, the pharmacogenetic studies from the mentioned Asian populations may not be representative of other types of Asian populations such as those from Central, Southern, and Southeast Asia. In addition, outcome measures such as opioid dose requirements, duration of treatment, and assessment of pain control implemented in the included studies were different, limiting the ability to compare findings between studies. Compared to pharmacogenomic studies in Caucasian and European populations, pharmacogenetic studies in Asian patients with cancer pain treated with opioids tend to have smaller sample sizes lacking adequate statistical power. Additionally, genetic association studies have focused on a limited number of genes and a few SNPs within each gene, which does not take into account other facets of pain manifestation such as a combination of SNPs (haplotypes), epigenetic factors, and drug-drug interactions, as well as other possible confounding factors. This review only included articles published in English, which may have resulted in publication bias, where some relevant non-English studies could have been missed.

This review provides insights into the association of genetic variants and treatment responses in Asian patients treated with opioids for cancer pain. The CYP2D6*10 SNP is relevant to tramadol pharmacokinetics and opioid dose requirement, while SNPs within *UGT2B7* (rs7439366), *ABAT* (rs1641025), and *P2RX7* (rs1718125) have demonstrated some level of potential clinical relevance to cancer pain pharmacogenomics in Asian patients. SNPs within *OPRM1*, *COMT*, and *ABCB1* were found to have low clinical significance due to several studies failing to replicate associations reported by others. Among these are the *OPRM1* rs1799971 implicated in morphine pharmacokinetics and pain control and *COMT* rs4680 implicated in morphine pharmacokinetics, opioid dose requirement, and pain control. These SNPs do not have consistent findings between Caucasian and Asian populations. Multiple *ABCB1* SNPs have been reported to have significance to opioid treatment. These include *ABCB1* rs1045642 associated with a dose of multiple opioids, rs1128503 associated with a dose of fentanyl and sufentanil, and rs2032582 associated with a dose of sufentanil. Further investigations are required to unravel the role of these SNPs in opioid treatment outcomes.

## 5. Conclusions

Inter-individual variability in opioid responses has been associated with polymorphisms in genes encoding drug-metabolizing enzymes (*CYP2D6*, *UGT2B7*), neurotransmitter-metabolizing enzymes (*COMT*, *ABAT*), transporters (*ABCB1*) and drug targets, including receptors (*OPRM1*) and ion channels (*P2RX7*). The clinical significance of pharmacogenetic biomarkers *OPRM1* rs1799971, *COMT* rs4680 and *ABCB1* (rs1045642, rs1128503, and rs2032582) should be further explored in future studies to improve precision pain management amongst Asian patients with cancer pain.

## Figures and Tables

**Table 1:** List of genes reported with significant associations in Asian populations.

Gene	Polymorphism(s)	Parameters with Significant Association	Ref.
*CYP2D6*	*10	Opioid dose requirements, pain control	[22,23]
	*2, *5, *10, *14	Pharmacokinetics	[24]
*CYP3A5*	*3	Pharmacokinetics	[25]
*UGT2B7*	rs7439366	Pharmacokinetics, pain control	[26]
		Pain control	[27]
*OPRM1*	rs1799971	Opioid dose requirements	[28]
		Pain control	[29]
		Opioid dose requirements	[30]
	rs1323040	Opioid dose requirements	[30]
*COMT*	rs4680	Opioid dose requirements, Pharmacokinetics	[31]
		Opioid dose requirements, pain control	[32]
*ABAT*	rs1641025	Opioid dose requirements, pain control	[33]
*ABCB1*	rs1128503	Opioid dose requirements	[25,30]
	rs2032582	Opioid dose requirements	[30]
	rs1045642	Opioid dose requirements	[34]
*P2RX7*	rs1718125	Opioid dose requirements	[35]

**Table 2:** Categorization of CYP2D6 gene phenotypes based on genotypes.

Normal metabolizer (NM)	*1*1, *1*2, *1*5, *1*10, *1*14, *2*2, *2*5, *2*10, or *2*14
Intermediate metabolizer (IM)	*5*10, *10*10, or *10*14
Poor metabolizer (PM)	*5*5, *5*14, or *14*14

Adapted with permission from [24].

**Table 3:** Polymorphisms significantly associated with opioid pharmacokinetics in cancer pain among Asian populations.

Gene (SNP)	Genotype (%)	Findings	*p*-Value	Population (n)	Opioid	Cancer Type	Ref.
*CYP2D6* (CYP2D6*2, *5, *10, *14)	NM ^1^ IM ^2^ PM ^3^	Plasma concentration of O-desmethyltramadol and its ratio to tramadol were lower in the CYP2D6 IM + PM group than in the n NM group.	<0.05	Japanese (70)	Tramadol	NA	[24]
*CYP3A5* (CYP3A5*3)	*1*1 (8.3) *1*3 (33.3) *3*3 (58.3)	Plasma concentration of fentanyl normalized with the measured absorption rate was higher in the CYP3A5*3/*3 group than in the *1/*1 and *1/*3 groups	0.048; 0.021	Japanese (60)	Fentanyl	Solid tumors	[25]
*UGT2B7* (C802T)	CC (13.33) CT (45) TT (41.67)	Plasma concentration of morphine for patients with CC genotype was significantly lower than that in patients with CT or TT genotype	0.05	Han Chinese (120)	Morphine	Solid tumors	[26]
*COMT* (G472A, rs4680)	GG GA AA	Plasma concentration of patients with AA genotype was lower compared to patients with AG and GG genotype on day 1	0.008	Japanese (48)	Morphine	Solid tumors	[31]

Abbreviation: SNP: single nucleotide polymorphism; n: sample size; NA: not available. ^1^ NM: normal metabolizer (*1*1, *1*2, *1*5, *1*10, *1*14, *2*2, *2*5, *2*10, or *2*14). ^2^ IM: intermediate metabolizer (*5*10, *10*10, or *10*14). ^3^ PM: poor metabolizer (*5*5, *5*14, or *14*14).

**Table 4:** Polymorphisms significantly associated with opioid dose requirements in cancer pain among Asian populations.

Gene (SNP)	Genotype (%)	MEDD (mg)	Findings	*p*-Value	Population (n)	Opioid	Cancer Type	Ref.
*CYP2D6* (CYP2D6*10)	Group I ^1^ (27) Group II ^1^ (41.3) Group III ^1^ (31.7)	459.5 ± 70.3 476.8 ± 99.2 532.7 ± 92.6	Tramadol consumption in group III was higher compared to group I or II at 4, 24, and 48 h. Tramadol consumption in group I and II did not differ.	<0.05	Chinese (70)	Tramadol	Gastric cancer	[22]
*CYP2D6* (CYP2D6*10)	WW ^2^ (21) WM ^2^ (54) MM ^2^ (25)	587.7 ± 132.0 600.4 ± 104.5 657.8 ± 185.2 (24 h after surgery)	Cumulative fentanyl consumption was higher in the MM group compared to the WW group at 6, 12, and 24 h postoperatively.	0.018	Chinese (207)	Fentanyl	Gastric cancer	[23]
*OPRM1* (rs1799971)	AA AG	29.97 51.37	Morphine mean dose decreased with age but increased with morphine treatment duration. Morphine dose requirements for patients with AG genotype was higher compared to patients with AA genotype.	0.043; 0.029 <0.001	Lebanese (89)	Morphine	Various ^3^	[28]
*OPRM1* (rs1799971)	AA (31) AG (58) GG (10)	NA	Tramadol/paracetamol combination treatment response was lower in patients with AG or GG genotypes than in patients with AA genotype. The requirement for rescue analgesia was also higher for patients with G allele variants.	<0.01 <0.01	Chinese (96)	Tramadol/PCM combination	Colorectal carcinoma	[29]
*OPRM1* (rs1799971)	AA (51) AG (35) GG (14)	64.35 ± 5.12 67.59 ± 4.89 71.52 ± 5.03 (24 h after surgery)	The consumption of sufentanil in the patient groups carrying the GG genotype was significantly increased compared to that of the AA and AG genotypes groups.	<0.05	Han Chinese (225)	Sufentanil	Lung cancer	[30]
*OPRM1* (rs1323040)	CC (55) CT (35) TT (10)	63.54 ± 4.87 67.71 ± 4.19 75.24 ± 3.98 (24 h after surgery)	The consumption of sufentanil in the patient groups carrying the TT genotype was significantly increased compared to that of the CC and CT genotypes groups.	<0.05	Han Chinese (225)	Sufentanil	Lung cancer	[30]
*COMT* (rs4680)	GG GA AA	43.0 ± 21.4 28.9 ± 3.2 30.0 ± 0.0	Morphine dose requirement of patients with AA genotype was lower compared to patients with AG and GG genotypes on day 1	0.03	Japanese (48)	Morphine	Solid tumors	[31]
*COMT* (rs4680)	GG (58) GA (38) AA (4)	35.2 ± 11.5 29.5 ± 2.3 25.0 ± 7.1	Morphine dose requirement of patients with GG genotype was higher compared to patients with AA and AG genotypes on day 1.	0.013	Japanese (50)	Morphine	Solid tumors	[32]
*ABAT* (rs1641025)	CC (59) CT (32) TT (8)	Before vs. after increased opioid dosage 1.9 ± 2.3 vs. 1.1 ± 1.5 6.5 ± 17.7 vs. 4.3 ± 16.4 8.0 ± 9.5 vs. 55.2 ± 100	Opioid dose requirement was lower in patient with CC genotype compared to patients with CT and TT genotype for both before and after increasing opioid dosage.	<0.001	Japanese (71)	Combination of opioids	NA	[33]
*ABCB1* (1236TT rs1128503)	CC (21.7) CT (40) TT (38.3)	NA	Rescue medication was needed by fewer patients with TT genotype compared to patients with CT and CC genotype	0.036	Japanese (60)	Fentanyl	Solid tumors	[25]
*ABCB1*(rs1128503	CC (58) CT (28) TT (14)	62.98 ± 5.68 67.89 ± 4.26 73.42 ± 3.97 (24 h after surgery)	The consumption of sufentanil in the patient groups carrying the TT genotype was significantly increased compared to that of the CC and CT genotypes groups.	<0.05	Han Chinese (225)	Sufentanil	Lung cancer	[30]
*ABCB1* (rs2032582)	GG (48.4) GA (37.8) AA (13.8)	NA	Sufentanil dose received was higher in patients with AA genotype compared to patients with AG and GG genotype at time points 6, 24, and 48 h.	<0.05	Han Chinese (225)	Sufentanil	Lung cancer	[30]
*ABCB1* (C3435Trs1045642)	CC (40.2) CT (43.7) TT (16.1)	NA	24 h and weight-surface-area-adjusted-24 h opioid dose received was higher in patients with TT genotype compared to patients with CC and CT genotypes.	0.057; 0.028	Han Chinese (112)	Various opioids ^4^	NA	[34]
*P2RX7* (rs1718125)	GG (46.2) GA (44.96) AA (8.82)	9.27 ± 3.06 10.45 ± 2.99 11.69 ± 3.40 (24 h post-operation)	Fentanyl dose requirement to control postoperative pain was higher in patients with GA and AA genotypes than in GG genotype.	<0.05	Han Chinese (238)	Hydromorphone	Lung cancer	[35]

Abbreviation: SNP: single nucleotide polymorphism; MEDD: morphine equivalent daily dosage; n: sample size; NA: not available; PCM: paracetamol; D1: Day 1. ^1^ Group I: without CYP2D6*10; Group II: heterozygous for CYP2D6*10; Group III: homozygous for CYP2D6*10. ^2^ WW (homozygous wild): CYP2D6*1*1; WM (heterozygous mutant): CYP2D6*1*10; MM (homozygous mutant): *CYP2D*6*10*10. ^3^ Gastrointestinal tract cancer; breast; lung; hematologic; urogenital; gynecologic; prostate; pancreas; head and neck; sarcoma; and others. ^4^ Codeine, fentanyl, hydrocodone, hydromorphone, levorphanol, methadone, morphine, oxycodone, oxymorphone, tramadol.

**Table 5:** Polymorphisms significantly associated with pain control in cancer pain among Asian populations.

Gene (SNP)	Genotype (%)	Findings	*p*-Value	Population (n)	Opioid	Cancer Type	Ref.
*CYP2D6* (CYP2D6*10)	WW ^1^ (21) WM ^1^ (54) MM ^1^ (25)	Pain score in the MM group was higher than in the WW group 6 h postoperatively	<0.05	Chinese (207)	Fentanyl	Gastric cancer	[23]
*UGT2B7* (rs7439366)	CC (13.33) CT (45) TT (41.6)	Pain score of patients with CT or TT genotypes were higher than patients with CC genotype	<0.05	Han Chinese (120)	Morphine	Solid tumors	[26]
*UGT2B7* (rs7439366)	CC (59.6) CT (31.6) TT (8.8	Pain score of patients with TT genotype was higher than patients with CC genotype	<0.05	Han Chinese (47)	Oxycodone	Tumors	[27]
*OPRM1* (rs1799971)	AA (31) AG (58) GG (10)	The difference in pre-treatment and post-treatment pain scores for patients with AA genotype was more significant than patients with AG or GG genotype. AA genotype patients showed lowered pain scores after receiving tramadol/paracetamol combination treatment	<0.01	Chinese (96)	Tramadol/PCM Combination	Colorectal carcinoma	[29]
*ABAT* (rs1641025)	CC (59.1) CT (32.4) TT (8.5)	Pain severity mean was lowest in patients with CT genotype compared to patients with CC or TT genotype after opioid dosage increment	<0.001	Japanese (71)	Combination of opioids	NA	[33]
*P2RX7* (rs1718125)	GG (46.2) GA (44.96) AA (8.82)	Carriers of the GA genotype had higher postoperative VAS scores than GG genotype carriers after general anesthesia and 6 h after surgery. Carriers of the AA genotype group had higher postoperative VAS scores than GG genotype carriers after general anesthesia, 6 h and 24 h after surgery	0.041; 0.030 <0.001; 0.006; 0.016	Han Chinese (238)	Fentanyl	Lung cancer	[4]

Abbreviation: SNP: single nucleotide polymorphism; n: sample size; NA: not available; PCM: paracetamol; VAS: visual analog scale. ^1^ WW (homozygous wild): CYP2D6*1*1; WM (heterozygous mutant): CYP2D6*1*10; MM (homozygous mutant): CYP2D6*10*10.

## Data Availability

Not applicable.
